# Microbiomes of Inflammatory Thoracic Aortic Aneurysms Due to Giant Cell Arteritis and Clinically Isolated Aortitis Differ From Those of Non-Inflammatory Aneurysms

**DOI:** 10.20411/pai.v4i1.269

**Published:** 2019-03-15

**Authors:** Ted M. Getz, Gary S. Hoffman, Roshan Padmanabhan, Alexandra Villa-Forte, Eric E. Roselli, Eugene Blackstone, Douglas Johnston, Gosta Pettersson, Edward Soltesz, Lars G. Svensson, Leonard H. Calabrese, Alison H. Clifford, Charis Eng

**Affiliations:** 1 Genomic Medicine Institute; Lerner Research Institute; Cleveland Clinic; Cleveland, Ohio; 2 Center for Vasculitis Care and Research; Department of Rheumatic and Immunologic Diseases; Cleveland Clinic; Cleveland, Ohio; 3 Center for Aortic Diseases; Heart Vascular Institute; Cleveland Clinic; Cleveland, Ohio; 4 Taussig Cancer Institute; Cleveland Clinic; Cleveland, Ohio; 5 Division of Rheumatology; Department of Medicine; University of Alberta; Edmonton, Alberta T6G 2R7, Canada; 6 Department of Genetics and Genome Sciences; Case Western Reserve University School of Medicine; Cleveland, Ohio; 7 Germline High Risk Focus Group; CASE Comprehensive Cancer Center; Case Western Reserve University School of Medicine; Cleveland, Ohio

**Keywords:** aorta, aneurysms, aortitis, microbiome, clinically isolated aortitis, giant cell arteritis

## Abstract

**Objective:**

We sought to characterize microbiomes of thoracic aortas from patients with non-infectious aortitis due to giant cell arteritis (GCA) and clinically isolated aortitis (CIA) and to compare them to non-inflammatory aorta aneurysm controls. We also compared microbiomes from concurrently processed and separately reported temporal arteries (TA) and aortas.

**Methods:**

From 220 prospectively enrolled patients undergoing surgery for thoracic aorta aneurysm, 49 were selected. Inflammatory and non-inflammatory cases were selected based on ability to match for age (+/-10 years), gender, and race. Biopsies were collected under aseptic conditions and snap-frozen. Taxonomic classification of bacterial sequences was performed to the genus level and relative abundances were calculated. Microbiome differential abundances were analyzed by principal coordinates analysis.

**Results:**

Forty-nine patients with thoracic aortic aneurysms (12 CIA, 14 GCA, 23 non-inflammatory aneurysms) were enrolled. Alpha (*P*=0.018) and beta (*P*=0.024) diversity differed between specimens from aortitis cases and controls. There were no significant differences between CIA and GCA (*P*>0.7). The largest differential abundances between non-infectious aortitis and non-inflammatory control samples included *Enterobacteriaceae, Phascolarctobacterium, Acinetobactor, Klebsiella*, and *Prevotella.* Functional metagenomic predictions with PICRUSt revealed enrichment of oxidative phosphorylation and porphyrin metabolism pathways and downregulation of transcription factor pathways in aortitis compared to controls. Microbiomes of aortic samples differed significantly from temporal artery samples from a companion study, in both control and GCA groups (*P*=0.0002).

**Conclusion:**

Thoracic aorta aneurysms, far from being sterile, contain unique microbiomes that differ from those found in temporal arteries. The aorta microbiomes are most similar between aneurysms that were associated with inflammation, GCA, and CIA, but differed from those associated with non-inflammatory etiologies. These findings are promising in that they indicate that microbes may play a role in the pathogenesis of aortitis-associated aneurysms or non-inflammatory aneurysms by promoting or protecting against inflammation. However, we cannot rule out that these changes are related to alterations in tissue substrate that favor secondary changes in microbial communities.

## INTRODUCTION

Aortitis is a clinical-histopathological diagnosis. Pre-operatively, it is often unrecognized; the diagnosis is usually made only after aortic aneurysm surgery and microscopic review [1]. Clinical and imaging evaluation is then required to determine diagnostic subsets of aortitis. Most forms of non-infectious aortitis have been presumed to be autoimmune and the result of a primary systemic large vessel vasculitis (LVV), such as giant cell arteritis (GCA) or Takayasu's arteritis (TAK), or other systemic autoimmune diseases (eg, rheumatoid arthritis, the spondyloarthritides, Behcet's disease, Cogan syndrome, relapsing polychondritis, systemic lupus erythematosus, Sjogren syndrome, granulomatosis with polyangiitis, sarcoidosis, or IgG4-related disease) [1, 2]. Aortitis may also appear in isolation, as a topographically limited lesion. This latter group has been the focus of recent consensus guidelines and coining the term “clinically isolated aortitis” (CIA) to indicate a focal-regional non-infectious form of vasculitis usually restricted to the proximal thoracic aorta [3, 4]. In any given case of CIA, it is uncertain whether vasculitis is pathologically truly isolated and potentially cured by surgical resection, or if it represents a more extensive vasculitis that is subclinical, undiscovered by imaging beyond the surgical site, and destined to be expressed as a systemic LVV in the future [3]. While some previous studies have found that patients with CIA are not at increased risk of future vascular events, most have found that many patients will develop new distal aortic aneurysms and/or other large vessel lesions [5, 6]. In a recent study of 196 patients with non-infectious aortitis, 66% had diagnoses of CIA, 21% had GCA, 7% had TAK, and 6% had other systemic inflammatory diseases, at the time of surgery. During a mean follow-up of over 4.5 years, 19% of patients with CIA developed new symptoms, 45% developed new radiographic vascular lesions, 40% underwent additional vascular surgery, and 12% died. Fifteen percent of patients initially classified with CIA developed features of a systemic disease, most often GCA [7]. Among numerous questions raised by such studies are the following: First, is CIA a limited presentation of diseases within a spectrum of LVV, including GCA? Second, do common conditions exist within CIA-affected and GCA-affected aortas and GCA-affected temporal arteries that favor developing inflammatory aneurysms or temporal artery inflammation and stenoses? And third, if this were the case, would those conditions differ from that present in non-inflammatory thoracic aortic aneurysms? Because of our interests in infectious triggers and vascular properties that favor the genesis of vasculitis, we examined the vascular microbiome in subsets of aortitis and non-inflammatory aorta aneurysm controls [8-11]. Unlike prior microbiome studies of large vessels, we collected surgically aseptic specimens that were snap-frozen and not fixed in formalin or paraffin-embedded.

## MATERIALS AND METHODS

### Sample Accrual and Collection

We prospectively enrolled 220 consecutive consenting patients undergoing thoracic aorta aneurysm surgery. Aortic samples were collected under strictly aseptic conditions. All aorta and temporal artery specimens (from a linked study [12]) were processed at the same time and analyzed in a blinded fashion.

The study protocol was conducted in compliance with the Helsinki Declaration and approved by the Institutional Review Board for Human Subjects' Protection at the Cleveland Clinic. Aorta samples were split into thirds, with one-third sent for routine histopathological review, one-third snap-frozen under sterile technique for microbiome analysis, and one-third utilized for a related biochemistry study [13]. Patients were classified according to clinical phenotype and histopathology as having either biopsy-positive and clinically compatible GCA (N=14) or clinically isolated aortitis (CIA, N=12), or non-inflammatory aneurysms (controls). Control aortas (N=190) were obtained from patients with aneurysms associated with diseases such as uncontrolled hypertension, bicuspid aortic valves, Marfan syndrome, and cystic medial degeneration of uncertain etiology. Controls (N=23) were selected to match aortitis cases (N=26) based on same gender, race and age (+/-10 years) (Table 1). Following >3 years of collection, all aorta and temporal artery samples, which were blinded to diagnosis, were processed at the same time.

**Table 1.** Demographic Characteristics, Comorbid Conditions, Substance Use History, and Relevant Laboratory Values of Patients with CIA, GCA, and Non-Inflammatory Aortitis Aneurysms.

Variables	CIA (#12)	GCA (#14)	Controls (#23)	P-value*
Age (yr) +/- SD	68.5+/-11.0	73.2+/-6.8	66.6+/-8.5	0.09
No. Female (%)	9 (75%)	13 (92.9%)	20 (87.0%)	0.44
BMI	29.2+/-2.1	29.9+/-1.9	27.0+/-1.3	0.39
No. White (%)	10 (83.3%)	14 (100%)	21 (91.3%)	0.25
No. Alcohol (%)	6 (50%)	5 (35.7%)	7 (30.4%)	0.6
No. Ever Tobacco (%)	11 (91.7%)	6 (42.9%)	11 (47.8%)	0.02
Hypertension (%)	9 (75%)	12 (85.7%)	14 (60.9%)	0.26
Hyperlipidemia (%)	8 (66.7%)	12 (85.7%)	10 (43.5%)	0.22
Coronary Artery Disease (%)	2 (16.7%)	6 (42.9%)	2 (8.7%)	0.05
WBC +/- SD	7.0+/-1.4	7.1+/-1.6	7.1+/-2.0	0.98
Hemoglobin +/- SD	13.0+/-1.8	14.3+/-7.3	13.3+/-1.6	0.71
Platelet Count +/- SD	220.1+/-71.1	232.8+/-70.1	224.4+/-66.2	0.89
Creatinine +/- SD	0.81+/-0.14	0.95+/-0.11	0.85+/-0.03	0.91
ESR>50 mm/hr (%)	0 (0%)	9 (64.3%)	0 (0%)	0.01
Concurrent systemic rheumatologic diseases	0 (0%)	0 (0%)	2 (8.7%)**	0.31
Steroid Use at Time of Surgery	0 (0%)	4 (28.6%)	0 (0%)	0.01

*Statistical analysis by 2-way ANOVA with *P*<0.05 considered significant. All quantitative values are shown as mean +/- standard deviation, and all qualitative values are shown as number of patients with characteristic (percentage of group in column with characteristic).

**One each with rheumatoid arthritis and Sjogren syndrome.

### 16S Ribosomal RNA Gene Sequencing of Aorta Biopsies

Total deoxyribonucleic acid (DNA) was isolated using the RNeasy PowerMag KitAllPrep Power-Viral DNA/RNA Isolation Kit according to the manufacturer's protocol (Qiagen, Valencia, CA) with minor modifications [14]. Briefly, all beads, tubes, and non-enzymatic reagents were treated with UV light for 30 minutes prior to use; samples were digested with 20 µL of 20 ng/µL Protein-ase K (Roche Diagnostics Corp., Indianapolis, IN) at 65°C degrees for 1 hour, then transferred to 0.1 mm glass bead-containing tubes, after which the samples were homogenized using the Tissue-Lyser LT (Qiagen). The quality and purity of the isolated total DNA were confirmed spectrophotometrically using a NanoDrop 2000 device (Fisher Scientific SAS, Illkirch, France). DNA concentration was quantified using the Qubit 2.0 instrument applying the Qubit dsDNA HS Assay (Life Technologies, USA). Extracted DNA samples were stored at -20°C.

Bacterial 16S rRNA gene amplification and library construction were performed according to the 16S Metagenomic Sequencing Library Preparation guide from Illumina (Forest City, CA). In brief, 2 µL total DNA was amplified using primers targeting the 16S V3 and V4 region (Illumina) at 95°C for 5 minutes, followed by 35 cycles of 95°C for 30 seconds, 72°C for 30 seconds, and a final extension of 72°C for 10 minutes. Then 16S rDNA amplicons were run out on a 1% agarose gel, size-selected at 450-500 bp, and gel-purified using QIAquick Gel Purification kit (Qiagen). A second round of PCR was performed to add Nextera XT indices (Illumina) to purified amplicons. Indexed PCR products were cleaned with Ampure XP beads (Beckman Coulter, Inc., Brea, CA) and resulting libraries quantified with the QuantiFluor dsDNA system according to the manufacturer's protocol (Promega, Madison, WI). Samples were then normalized to 10nM and pooled into sequencing libraries. Pooled V3-V4 amplicon libraries were sequenced using the Illumina MiSeq platform with V3 reagent kit. The 300-bp paired-end reads for each sample were demultiplexed and quality checked using FastQC 0.11.3.

Because the specimens were stored in aluminum foil and sterile containers, 16S rRNA sequencing was performed from both and revealed identical results. The containers had sterilized water ali-quoted into them which was then utilized as a template for 16S rRNA gene sequencing, revealing no contamination.

### Microbiome Analysis

All microbiome analysis was performed in a blinded fashion, in the first instance, after which a co-author not involved in the microbiome analysis unblinded meta-data.

A hybrid post-sequencing analysis methodology using QIIME and MICCA was adopted, in which preprocessing was performed in QIIME and open-reference operational taxonomic unit (OTU) picking was performed with MICCA and Phyloseq [15]. After the biom files were created, downstream analysis was performed with QIIME. Then 250-bp Illumina Paired-end reads were merged with FLASH [16] and low quality reads (Phred < 20) were filtered out using the split_libraries.py command in QIIME (version 1.9) [17]. MICCA vsearch (version 1.9.5)[18] was utilized for clustering the sequences with a threshold of 97% similarity, and representative sequences were classified using RDP classifier (version 2.11) [19]. Multiple sequence alignments were done on the comprehensive sequence using MUSCLE (version 3.8.31) [20, 21] against the Greengenes database (version 13.8) [22], filtered at 97% similarity, and FastTree (version 2.1.8) was used for phylogenetic tree construction [23].

Data clean-up was performed by removing the singletons and discarding taxa represented in fewer than 5% of total samples. The rarefaction value was set to 1107 reads per sample to reduce sampling heterogeneity, and computation of alpha (Shannon diversity index) and beta diversity measures (unweighted UniFrac distances) were performed with phyloseq in R. Alpha diversity measures species richness (number of taxa) within a single microbial ecosystem. Beta diversity can be represented by UniFrac distances which describe similarities and dissimilarities between bacterial communities using phylogenetic information, taking into account number of taxa and relative abundances within each taxon (Table 2). F-tests based on sequential sums of squares derived from 1,000 permutations on UniFrac distance matrices were performed with the null hypothesis that there is no difference in community structure between groups. Note that PCoA and the calculation of *P* values are measurements of clustering strength. Differences (and the *P* value) are derived from measuring differences between UniFrac distances. To find which taxa are most likely to explain the differences between our clinical groupings, taxa summaries and differential abundances were analyzed with DESeq2 with data from MICCA input into the R package DESeq2. This algorithm estimates variance-mean dependence in count data and tests for differential expression based on a model using the negative binomial distribution. Differentially abundant taxa that were statistically significant using an alpha of 0.01 and exceeded a log2-fold change of ±2 were visually represented on box plots. Comparisons between aorta and temporal artery micro-biomes were performed using microbiome dataset collected from a parallel study of 47 temporal arteries with and without GCA [12]. However, both the aorta and temporal artery samples were sequenced at the same time to reduce batch effect.

**Table 2.** Measures of Microbial Diversity as Applied in this Study

	Definition	Method of Quantification
Alpha	Degree of taxa* diversity (OTU**) within a sample (all temporal arteries or aortas or subsets) without regard to specific organisms present	Shannon index: H' = -∑^s^_i_=_1_(p_i_^ln^(p_i))_ Where p_i_ is the fraction of total species comprised by species *i*.
Beta	Degree of microbial similarity (or dissimilarity) between 2 samples [eg aorta aneurysms – aortitis (or subsets) vs. non-inflammatory disease] taking into account specific taxa present	Unifrac distance: Measures the phylogenetic distance between sets of taxa in a phylogenetic tree as the fraction of the branch length of the tree that leads to descendants from either one environment or the other, but not both.

* Genus or species

** OTU = operational taxonomic units

We predicted functional composition of microbiomes using the PICRUSt 1.0.0-dev bioinformatics package [24]. We filtered out all de novo OTUs and used this OTU table as our input into the PICRUSt algorithm, which calculates contributions of various OTUs to known biological pathways based on evolutionary modeling. Welch's *t* test was used to calculate *P* values, and corresponding Storey *q-*values were used to control for the false discovery rates associated with multiple testing. These values were calculated using DESeq2 and LEfSe and visualized as a bar plot using STAMP [25].

## Results

### Patients

Twenty-six patients with aortitis-associated thoracic aneurysms, including 14 with GCA and 12 with CIA, and 23 controls with non-inflammatory aorta aneurysm, were enrolled in our study (Table 1). There were no differences in demographics, exposures, and relevant past history among the 3 groups, except for tobacco use (*P*=0.02), a history of coronary artery disease (*P*=0.05), and steroid use in 4/14 patients with GCA (Table 1). Importantly, there was no significant difference in concurrent systemic autoimmune disease across the groups (*P*=0.31, Table 1). Nine of 14 patients with GCA had an ESR>50 mm/hr compared to none in both the CIA group and controls (*P*=0.01, Table 1). Other basic laboratory values did not differ across the 3 groups (*P*>0.7, Table 1). Additionally, none of the patients were taking antibiotics at the time of their surgical procedures.

### Histopathology

Of the 26 eligible patients with aortitis, 14 received a final diagnosis of biopsy-proven GCA revealing typical arteritis with mononuclear cell inflammatory infiltrates localized to the adventitia and media, giant cell granulomatous changes (9/14), fragmentation of the internal elastic lamina, and varying degrees of intimal proliferation and fibrinoid necrosis. The remaining 12 were diagnosed with CIA with giant cell granulomatous changes (8/12), a lymphoplasmacytic pattern of inflammation, a suppurative pattern of inflammation, or a mixed inflammatory pattern. Among the 23 control aortas, 21 had diagnoses that are considered to contribute to thoracic aorta aneurysm formation including bicuspid aortic valve (8), atherosclerosis with calcification of the aortic valve (5), cystic medial degeneration of uncertain etiology (3), hypertension (14), and Marfan syndrome (1).

### Microbiome in Aortas From Patients With and Without Aortitis

**Figure 1.**
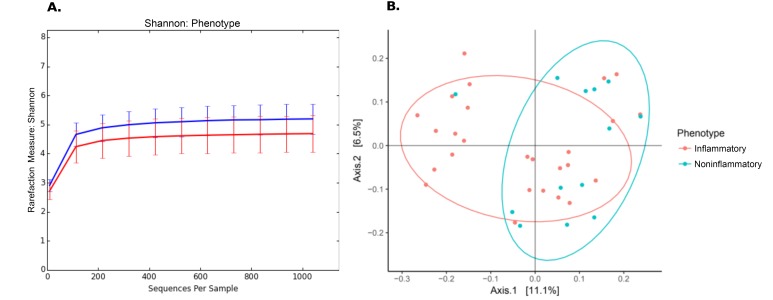
**Differences in microbiome in aortas from patients with aortitis compared to those without aortitis.** (A) Rarefaction (Shannon diversity) curves showing decreased alpha diversity in aortitis samples (red curve) compared to control aortas (blue) [*P*=0.018]. (B) Principal component analysis (PCoA) of aorta microbiomes from patients with aortitis (red) compared to those from control patients (green) [*P*=0.024].

Culture-independent, long-read genomic sequencing was used to characterize the entire microbial communities of aortas from the 49 research participants with aorta aneurysm. After sequencing and quality control, 10 samples, all from controls, were excluded from further analyses due to low read count, leaving all 26 samples with aortitis and 13 controls. Alpha diversity (Table 2) was reduced in aortitis samples compared to control aortas (*P*=0.018, Figure 1A). Beta diversity (Table 2), as measured by UniFrac distances, differed in aortitis samples compared to controls (*P*=0.024, Figure 1B). Importantly, there were no differences in alpha or beta diversity between GCA aorta samples and CIA aorta samples (*P*>0.7, data not shown).

Irrespective of diagnosis, there was borderline decreased beta diversity in aortas from patients with a history of hypertension compared to normotensive patients (*P*=0.05), which disappeared when microbiomes were compared within the aortitis group (*P*=0.2) and within the control group (*P*=0.7). Of note, the histories of hypertension were not different between aortitis cases and controls (Table 1).

**Figure 2.**
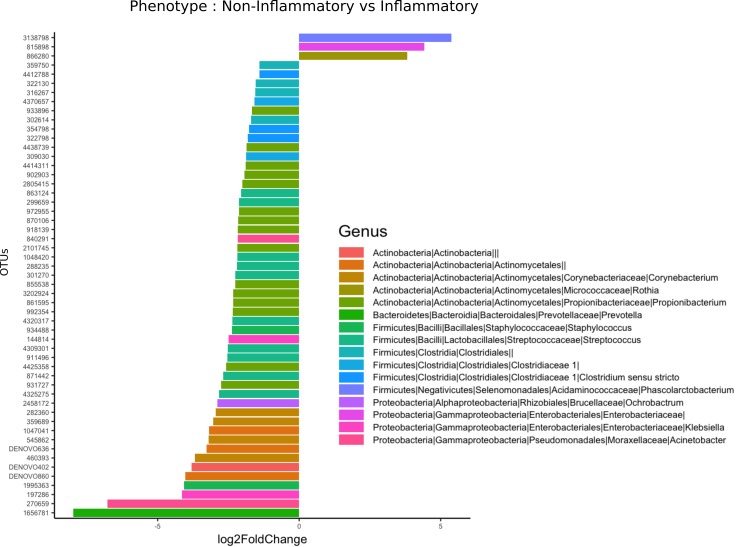
**Most differentially abundant taxa in aortas from patients with aortitis and from control patients.** Bar plot representation showing the most (and statistically significant, all *P*<0.05) over-represented (+) (right) and under-represented (-) (left) taxa in aortas from aortitis patients compared to those from control patients.

We then examined taxonomic classifications and relative abundances of bacterial species within aorta samples in order to determine whether differential taxon abundances exist in aortitis specimens compared to non-inflamed control aortas. At the phylum level, OTUs from Actinobacteria and Firmicutes were relatively less abundant in aortitis specimens compared to control aortas (Figure 2). An OTU from the genus *Phascolarctobacterium* had the greatest over-representation in aortitis samples compared to control aortas, followed by OTUs from the family *Enterobacteriaceae* and the genus *Rothia* (Figure 2). The most differentially less abundant OTU in aortitis compared to controls was the genus *Prevotella*, followed by the genera *Acinetobacter, Klebsiella, Staphylococcus*, and *Cornybacterium*. There were also 7 less abundant OTUs that were unclassified at the genus level from phylum Actinobacteria, 15 belonging to the genus *Propionbacterium,* 1 to the genus *Onchrobactrum*, and 9 to the class Clostridales (Figure 2).

### Predicting Functional Consequences of Differing Microbial Composition in Aortitis Specimens and Control Aortas

**Figure 3.**
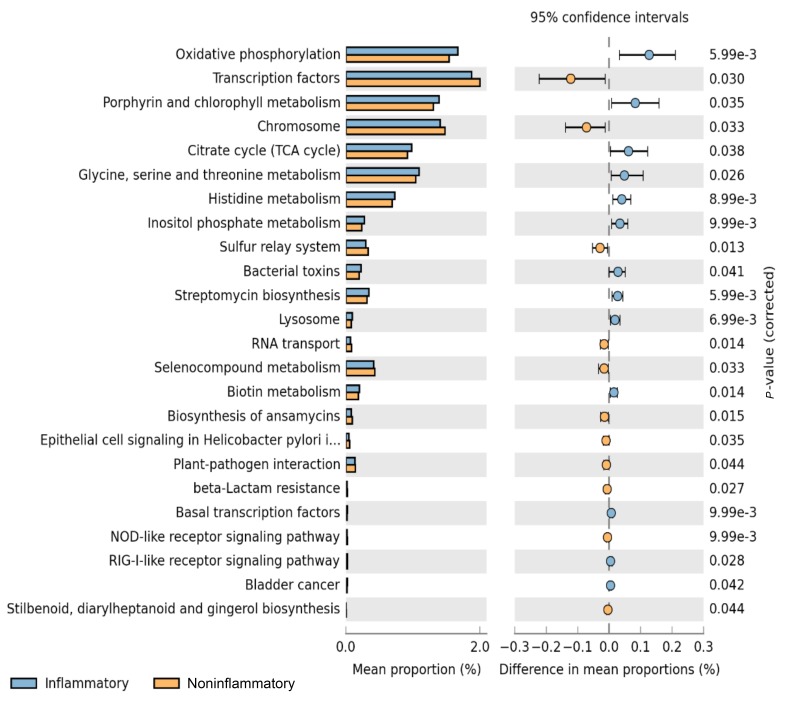
**Predicted functional pathways differentially represented in aorta samples from patients with aortitis compared to those from controls.** Representation of PICRUSt analysis yielding differentially regulated functional pathways in aortas from cases (blue bars) and controls (orange bars).

Metagenome functional content was predicted using PICRUSt, and group comparisons between the aortitis and control groups were performed with STAMP (Figure 3). With the different microbes in aortitis samples compared to control aortas as input, PICRUSt predicted relative upregulation of pathways involved in oxidative phosphorylation, porphyrin metabolism, TCA cycle, streptomycin biosynthesis, and glycine, serine, threonine, and histidine metabolism in aortitis. In contrast, predicted relative downregulated pathways included transcription factors, chromosome maintenance, the sulfur relay system, and selenocompound metabolism.

### Comparison of Microbiomes in Aortas and Temporal Arteries

**Figure 4.**
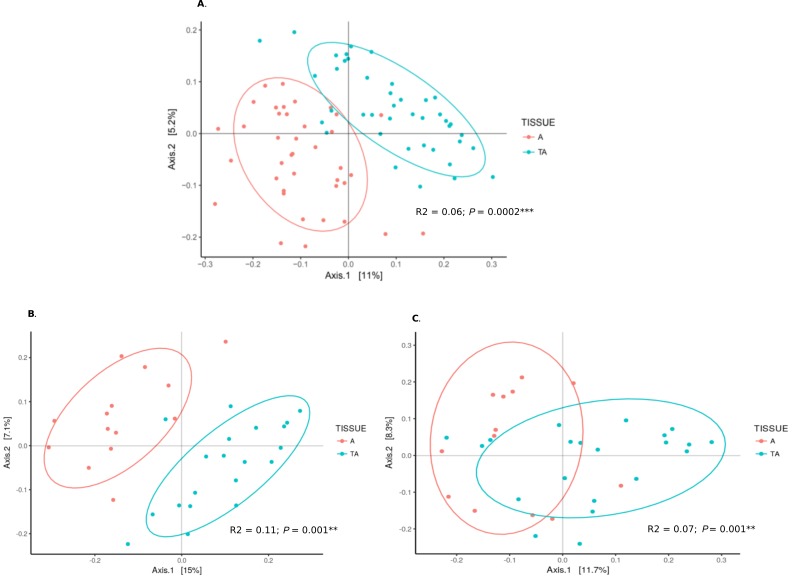
**Microbiome from aortas compared to temporal arteries.** (A) PCoA of microbiomes from all aorta specimens (red) and all temporal arteries (green). (B) PCoA of microbiomes from control aortas (red) and control temporal arteries (green). (C) PCoA of microbiomes from aortitis specimens (red) and temporal arteritis samples (green).

Gene sequencing data of 16S rRNA were compared between our 49 aorta samples and 47 temporal arteries, the latter from a parallel study [12], 23 without GCA and 24 with GCA. There was a significant difference in beta diversity between the microbiome of aorta and temporal arteries (*P*=0.0002, Figure 4A). Microbiomes from GCA-affected temporal arteries [12] versus GCA-affected aortas were significantly different (*P*=0.001), as was also noted for the non-inflammatory aortas and non-inflammatory temporal artery controls (*P*=0.001). Of note, the separation by UniFrac distances by PCoA was qualitatively more marked in control temporal arteries versus control aortas (Figure 4B, almost complete separation) compared to those of temporal artery-GCA and aortitis-GCA (where there is overlap, Figure 4C).

## Discussion

Our results suggest that, far from being sterile, aortas contain a microbiome in both inflammatory and non-inflammatory states. Interestingly, we found that the aortic microbiome of patients with inflammatory conditions differed substantially from our controls in both alpha diversity and beta diversity. However, we found no difference in diversity between the LVV subsets, GCA and CIA, within our aortitis group. Additionally, the observations that the histopathology of GCA and CIA are indistinguishable and that a significant minority of patients with clinically isolated aortitis phenotype may evolve into a giant cell arteritis phenotype [10], raise questions about shared critical pathogenetic mechanisms for these entities. Conversely, we do not know whether the inflammatory state and/or its consequences observed in these forms of LVV could impact the composition of the observed microbiome. Alternatively, these microbial differences could be influenced by variables not examined in these studies.

Prior studies of GCA pathogenesis have focused on temporal arteries. *Mycoplasma pneumonia, Chlamydia pneumonia*, and *Burkholderia pseudomallei-like* organisms [26-28] have been implicated in pathogenesis; however, none have been confirmed by others nor were they detected in our aorta dataset or in a separate study we performed on temporal arteries [12].

Not surprisingly, we found that the microbiomes of temporal arteries and aortas differed both between the GCA-affected temporal arteries and aortas, and the non-inflammatory aortas and non-inflammatory temporal artery controls. There are many precedents for the same stimuli having a range of effects in different tissues and even territories within a shared network (eg, the vasculature). Consider unique site predilections in atherosclerosis (eg, the infra-renal aorta) and even infection-mediated vasculitis (eg, hepatitis C, preferred targeting of skin, kidneys, and peripheral nerves). Disease targeting or sparing even within the same vessel (eg, proximal and distal aorta) may differ for many reasons including differences in embryogenic origins of smooth muscle cells within the aortic root and arch versus the more distal aorta, gene expression, and focal immune response [8].

The most thorough survey of the temporal arterial microbiomes so far examined the role of microbes in GCA [29]. Bhatt and colleagues utilized a whole genome sequencing approach to search for a pathogen in 17 formalin-fixed, paraffin-embedded temporal artery samples. More than 1000 non-human microbial species were detected; however, by far the most common were *Propionibacterium acnes* and *Escherichia coli*, which are commonly found within human skin flora. Moreover, these authors did not detect any significant differences between GCA-affected temporal arteries versus controls in regard to the relative abundance of bacterial species nor did they find any previously reported viral pathogens. Bhatt et al provided insight into the spectacular diversity of the vascular microbiome, but their study suffers from its use of non-sterile collection methods and the use of formalin-fixed, paraffin embedded tissue [29]. Consistent with these observations, we found that *Proprionibacterium* are common in the vasculature. Moreover, we found signifi-cant differences in the microbiomes of aortitis and control vessels. Our study had the advantage of using both aortic tissue and temporal arteries [12], as well as using fresh frozen samples collected aseptically, which we believe allows for a more accurate representation of constituents of the vascular microbiome, while minimizing microbial contaminants. Importantly, all samples and meta-data were blinded during preparation, sequencing and microbiome/bioinformatics analyses. That the vascular microbiome differs between the 2 conditions studied, inflammatory and non-inflammatory aorta control specimens, processed at the same time, also argues strongly against contamination.

In this study, we not only found distinct microbial communities in our aortitis and control groups, but also found that the aortitis specimens had significantly less phylogenic diversity than our control specimens. The finding that decreased microbial diversity may promote inflammation is well established. For example, in inflammatory bowel disease (IBD), a disease for which there is an extensive microbiome literature, among the most consistent findings is a reduction in overall microbial diversity, including species that are thought to have anti-inflammatory properties [30]. Decreased bacterial diversity has been associated with other rheumatologic diseases, even in areas not traditionally thought to contain a microbiome. A recent study of 30 patients with early rheumatoid arthritis (RA) or sarcoidosis, found that the lungs of patients with rheumatologic disease had distal airway dysbiosis with 40% fewer OTUs when compared to the microbiome of healthy controls [31]. Thus, 1 hypothesis that may be derived from our results is that rather than aortitis being incited by a single or a few pathogenic strains, bacterial diversity may actually confer protection against inflammation and loss of important commensals may promote disease susceptibility.

Our findings included a relative over-representation in the genera *Phascolarctobacterium* and *Rothia* and the family *Enterobacteriaceae* in the aortitis group. All of these are ubiquitous components of the human skin and gastrointestinal flora and known human pathogens with the potential to drive inflammation. *Enterobacteriaceae* has been implicated as a trigger of inflammation in both IBD and colon cancer and has been demonstrated to activate the inflammosome, promoting pro-inflammatory cytokine secretion [32]. Some of the over-represented genera have the potential to become pathogenic, but they do not dominate as would be expected with a frank bacterial infection; rather, they form a general pro-inflammatory milieu, which may contribute to the development of aortitis.

Interestingly, we found numerous OTUs were under-represented in our aortitis group when compared to the control aortas, consistent with the observed decrease in overall microbial diversity in the aortitis group. These included the genera *Prevotella, Acinetobacter, Klebsiella, Staphylococcus*, and *Cornybacterium*, many of which have been found to be protective against inflammation on mucosal surfaces. Additionally, *Klebsiella* and *Staphylococcus* have been previously isolated from atherosclerotic plaques and are known to form biofilms, allowing for adherence to vessel walls [11]. *Prevotella*, a GI commensal, is commonly observed to be elevated in several inflammatory conditions; however, it has also been found to be less frequent in inflammatory airway diseases such as asthma and chronic obstructive pulmonary disease [33, 34]. Similarly, 1 study found that increasing abundance of *Prevotella* was associated with lower levels of inflammatory cytokines IL-10, IL-23, and TNF-α in healthy lungs [34]. *Acinetobacte* has also been found to be protective against airway inflammation in asthma, through suppression of Th2 mediated T-cell responses to allergens [35]. Together, the reduction in these OTUs may have the effect of facilitating inflammation via loss of anti-inflammatory metabolites and host-microbe interactions with the immune system.

Prediction of up and downregulation of functional pathways based on the composition of the microbiome revealed a relative increase in oxidative phosphorylation and TCA cycle pathways in the aortitis group. One possible explanation for this is that it reflects an increase in aerobic and facultative aerobic microbes, including *Enterobacteriaceae* and *Rothia*. Porphyrins are a group of metabolites that have previously been linked to inflammation in acne via production of reactive oxygen species [36]. Several microbial species have been found to increase the secretion of porphyrins and upregulate enzymes responsible for porphyrin metabolism when exposed to hemin, an iron containing porphyrin found in hemoglobin [37, 38]. Consistent with these reports, we found that porphyrin metabolism was predicted to be upregulated in our aortitis group. It is tempting to speculate that these porphyrin metabolites may play a role in inducing inflammation in aortitis. Understanding the predicted downregulation in pathways responsible for chromosomal maintenance and transcription factors is more opaque, but it is possible that these pathways reflect decreased microbial replication in the setting of immune activation. However, further studies are required to clearly establish a firm relationship between the predicted up and down-regulation of pathways and the microbes we detected in the inflammatory and non-inflammatory aorta samples.

The notion of a vascular microbiome is neither new nor novel [11]. Many studies have demonstrated the presence of both bacteria and viruses within the walls of blood vessels. The best evidence for the role of bacteria in vascular pathology comes from the isolation of microbes from atherosclerotic plaques, including *C. pneumoniae, S. epidermidis, P. acnes, P. gingivalis, P. granulosum, Micrococcus* sp., and *A. viscosus* [39, 40]. Moreover, the discovery of T cells specific for *C. pneumoniae* and *P. gingivalis* implicate microbes in the pathogenesis of atherosclerosis as does the finding that mice inoculated with *P. gingivalis* experienced accelerated atherosclerosis [41-43].

Recent studies have also lent support for the idea that apparently normal vessels may contain a stable microbiome. One of the most compelling of these studies applied a 16S-metagenomics sequencing approach to 56 fresh, sterile aortic aneurysm samples from patients with atherosclerosis and non-atherosclerotic disease. Bacterial DNA was isolated from about 90% of samples, and 10 samples were selected for speciation [39]. All but 1 specimen revealed the presence of multiple bacterial species. Similarly, a study of healthy femoral and coronary arteries in bypass grafts, using 16S sequencing, found 245 bacterial species and 90 genera, including *Streptococcus*, which was a major component of both the control and aortitis microbiome in our investigation [44].

The major limitations of our study are relatively small sample size and the inability to procure sterile healthy aortas as controls. For controls, we utilized samples from patients undergoing aorta reconstruction for aneurysms caused by non-inflammatory conditions such as hypertension or congenital/genetic anomalies. While we found that our non-inflammatory aorta aneurysm controls differed significantly from the aortitis-aneurysm samples, it is possible that our controls differ from healthy vessels in unforeseen ways. However, our study did have several important strengths. It is the first to be performed in a blinded fashion on surgically sterile aortas that were maintained under strict aseptic conditions throughout processing, reducing the possibility of contamination. Moreover, we used aortitis samples from 2 forms of LVV (GCA and CIA) that are histologically indistinguishable and may be pathogenically related, and compared their microbiomes to non-inflammatory aortic aneurysm controls.

In conclusion, our results emphasize that thoracic aorta aneurysms are not sterile but contain microbial populations regardless of diagnosis. These microbiomes differ between topographically distinct GCA-affected aortas and temporal arteries. While that could argue against specific microbial agents in these populations having an etiologic role, it is possible that these distinct populations could foster changes that have similar pathogenetic effects. Additionally, the microbiomes of patients that have aortitis have significantly less phylogenic diversity than our controls, and they cluster into a distinct group when compared to controls. Aortitis cases also had an increased prevalence of known pathogens that may contribute to a pro-inflammatory environment. While these findings imply possible roles for microbiota in LVV, further studies are needed to elucidate whether the observed differences in microbial populations in idiopathic aortitis versus non-inflammatory aneurysms are pathogenic or secondary to vessel substrate injury.
